# A Multi-Scale Object Detection Network with Integrated Spatial-Channel Collaborative Attention for Remote Sensing Images

**DOI:** 10.3390/s26041370

**Published:** 2026-02-21

**Authors:** Lijun Ma, Chengjun Xu, Kun Jiao, Wenming Pei, Hongfei Zhang, Lanfeng Liu, Bin Deng, Juan Wu

**Affiliations:** 1 College of Energy (College of Modern Shale Gas Industry), Chengdu University of Technology, Chengdu 610059, China; 2 School of Remote Sensing and Information Engineering, Wuhan University, Wuhan 430072, China; 3 Nanjing Institute of Environmental Sciences, Ministry of Ecology and Environment, Nanjing 210042, China

**Keywords:** multiscale feature extraction, remote sensing images, cross-attention mechanism, deep learning, computational efficiency

## Abstract

**Highlights:**

**What are the main findings?**

- The proposed model, which integrates a novel cross-channel multi-scale feature extraction (CC-MSFE) module and a channel-spatial cross-attention (CSCA) mechanism, achieves good performance on three public remote sensing datasets (DIOR: 78.1% mAP, HRRSD: 90.6% mAP, and RSOD: 96.5% mAP).
- The framework effectively performs multi-scale object detection within complex environments, achieving enhanced accuracy while reducing computational complexity (Parameters: 19.5 M, FLOPs: 75.2 G).

**What are the implications of the main findings?**

- It provides a practical and efficient solution for multi-scale object detection in complex remote sensing scenarios.
- This innovative cross-attention structure design provides practical guidance for other feature extraction scenarios in remote sensing applications.

**Abstract:**

In remote sensing object detection, current models typically employ feature extraction modules and attention mechanisms to tackle issues such as significant scale variations among targets, cluttered backgrounds, and the subtle characteristics of small objects. Nevertheless, existing feature extraction approaches often depend on convolution kernels with fixed sizes, which can blur the contours of large objects and provide inadequate feature representation for small objects. Moreover, many attention mechanisms simply combine spatial and channel attention, without fully considering the deep integration between spatial and channel features, consequently leading to high-dimensional features and considerable computational overhead. To overcome these shortcomings, this paper introduces a multi-scale object detection network with integrated spatial-channel collaborative attention for remote sensing images. This approach enhances feature perception and representation for multi-scale targets, particularly small targets, through the design of the cross-channel multi-scale feature extraction module (CC-MSFE). Furthermore, a new channel-spatial cross-attention mechanism (CSCA) is introduced, comprising the channel attention mechanism (CA), the spatial attention mechanism (SA), and the cross-attention fusion module (CAFM). This design fosters dynamic interaction and joint optimization across channel and spatial dimensions, thereby improving detection accuracy while effectively reducing computational cost. The efficacy of the proposed model is evaluated on three publicly available remote sensing datasets. Experimental results show that the model achieves a mAP of 78.1% on the DIOR dataset and of 90.6% on the HRRSD dataset, outperforming YOLOv11 by 0.7% and 1.4%, respectively. On the RSOD dataset, it attains a mAP of 96.5%, surpassing YOLOv8 by 2.1%. In addition, the proposed method maintains a notably lower parameter count and computational complexity compared to existing approaches, achieving an effective balance between detection accuracy and computational efficiency.

## 1. Introduction

**Figure 1:**
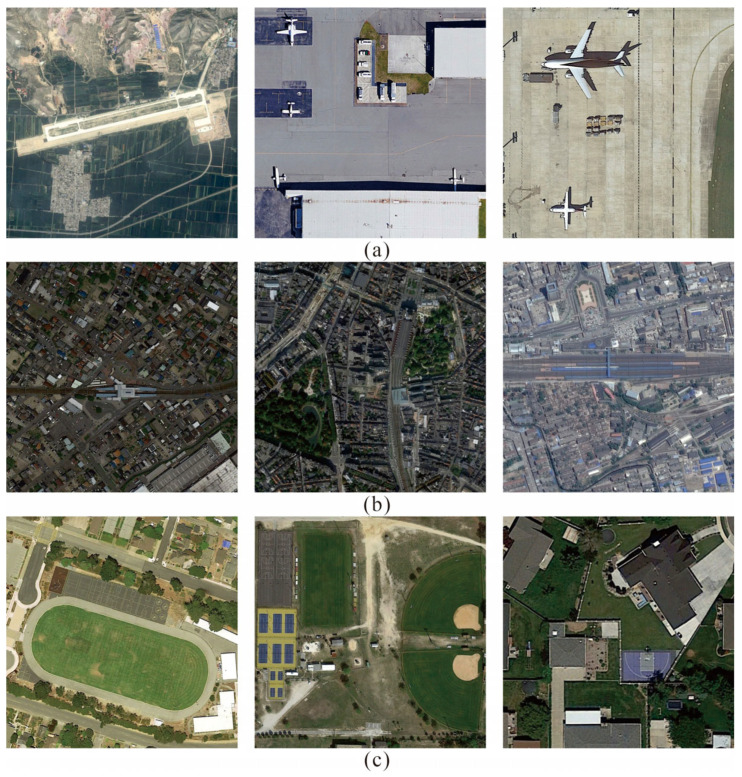
Typical challenges in remote-sensing imagery: (**a**) extreme scale variation; (**b**) complex backgrounds; (**c**) high degree of similarity between categories. The above image is from the DIOR dataset.

Remote Sensing Object Detection (RSOD) aims to automatically identify and locate target objects (such as vehicles, ships, aircraft, etc.) from remote sensing images captured by aviation or satellites [1,2]. It is extensively utilized in various sectors, including resource monitoring [3,4], urban development [5,6] and military reconnaissance [7]. With the rapid advancement of sensor technologies, modern remote sensing images are characterized by ultra-high spatial resolution and extensive coverage, providing abundant visual details [8,9,10]. However, this progress also introduces substantial challenges for object detection, especially for small and densely distributed targets. In addition, remote sensing imagery typically exhibits extreme scale variation, complex and cluttered backgrounds, arbitrary object orientations, and high inter-class similarity, as shown in Figure 1. Small objects often occupy only a few pixels and present weak visual cues, making them easily confused with background textures or suppressed by noise and shadow effects. These characteristics severely limit detection accuracy and robustness, and have become a major bottleneck in fully exploiting the value of large-scale remote sensing data [11].

Initially, object detection based on traditional methods depended on hand-crafted features [12,13], such as Histogram of Oriented Gradients (HOG) [14] and Bag-of-Words (BoW) [15], combined with traditional machine learning classifiers. Although such approaches laid important foundations, their limited representation capacity and dependence on expert-designed features make them difficult to generalize to complex real-world remote sensing scenes. With the emergence of deep learning [16], convolutional neural networks (CNNs) have significantly improved detection performance by learning hierarchical features in an end-to-end manner. For example, two-stage detectors such as Faster R-CNN [17] and one-stage detectors such as SSD [18] and YOLO [19] have become representative object detection frameworks, and have also been widely adopted and extended in remote sensing scenarios. Nevertheless, existing deep learning–based detectors still face notable limitations when applied to remote sensing imagery. In particular, CNNs are relatively limited in explicitly modeling long-range dependencies, and their feature representations are often insufficient for accurately capturing small objects embedded in complex backgrounds.

To address extreme scale variation and improve small-object representation, multi-scale feature extraction and fusion have become central components in modern RSOD frameworks. By aggregating information from different receptive fields and feature levels, multi-scale representations can enhance robustness to object size changes and enrich contextual cues. However, existing multi-scale feature extraction methods, such as Feature Pyramid Network (FPN) [20] and its variants like Dense Feature Pyramid Network (BDFPN) [21], primarily emphasize spatial-scale fusion across pyramid levels, while the interaction among feature channels is largely overlooked. Treating channels independently limits the network’s ability to capture cross-channel semantic correlations and to exchange complementary information across scales, which is crucial for small and densely distributed objects with weak and ambiguous visual patterns.

In recent years, attention mechanisms have been introduced into remote sensing object detection to enhance feature discrimination and improve long-range dependency modeling. Representative RSOD methods, such as SCRDet [22] and RT-DETR [23], incorporate attention mechanisms to suppress background interference and highlight target regions. Despite their effectiveness, most existing attention-based approaches treat channel attention and spatial attention independently or simply combine them in a sequential manner. Such designs fail to fully exploit the intrinsic correlation between channels and space, often leading to redundant features, increased computational cost, and suboptimal performance for small or multi-scale objects.

Moreover, many state-of-the-art RSOD models adopt complex network architectures with a large number of parameters, which hampers their deployment in practical scenarios where computational resources are limited. Therefore, achieving a favorable balance between detection accuracy and computational efficiency remains a critical challenge in RSOD.

Based on the above analysis, we propose a lightweight multi-scale object detection network with integrated spatial–channel collaborative attention. By performing cross-channel feature extraction and deeply coupling channel attention with spatial attention, the proposed method aims to strengthen feature discrimination for multi-scale object detection, especially for small objects in complex and cluttered backgrounds, while maintaining high computational efficiency.

The main contributions are summarized as follows:

(1) We propose a new cross-channel multi-scale feature extraction module (CC-MSFE), which facilitates the interaction of features across channels through the cross-fusion of various convolutional channels. This approach enhances the holistic perception of multi-scale target feature information and effectively extracts local detail information.
(2) We propose a new channel-spatial cross-attention mechanism (CSCA). This mechanism first innovatively designs channel attention and spatial attention, which effectively retain key channel and spatial information while improving the context awareness ability. Furthermore, the cross-attention fusion module (CAFM) is utilized to deeply integrate the two attention mechanisms, achieving complementary features between channels and spaces, effectively reducing redundant features, and thereby reducing the computational burden of the model.
(3) Experimental results on the DIOR, HRRSD and RSOD datasets show that the model significantly enhances detection precision for multi-scale objects in complex backgrounds, while reducing the number of parameters and computational complexity, achieving an efficient lightweight design.

## 2. Related Work

### 2.1. Remote Sensing Object Detection Based on CNN

In 2014, Girshick et al. [24] proposed R-CNN, which was the first to apply convolutional neural networks to the detection task and achieved nearly a 30% improvement in detection accuracy. This technique depends on the neural network for the automatic and efficient extraction of target feature information from images, enabling the rapid development of deep learning-based target detection technology. It avoids the errors caused by manually designed features, achieves higher accuracy and shorter detection time than traditional methods, and promotes the transformation of target detection tasks from traditional methods to intelligent, end-to-end learning methods.

RSOD based on deep learning can be divided into two categories: two-stage detectors and single-stage detectors [13]. The two-stage detectors divide the detection process into two steps. The first step is to generate candidate regions that may contain the target, and the second step is to classify each candidate region and perform precise bounding box regression. The R-CNN series of models enhances the efficiency and functionality of target detection through key innovations: R-CNN first applied CNN to detection, but with low efficiency. Fast R-CNN [17] significantly improved the training and detection speed on the basis of R-CNN through RoI Pooling and end-to-end training. The Faster R-CNN [25] algorithm introduced the region proposal network (RPN), which greatly improved the generation speed of candidate boxes and the overall detection efficiency. Mask R-CNN [26] first combined object detection with semantic segmentation, leading to a certain improvement in detection accuracy. While the R-CNN series of algorithms has enhanced the accuracy and reliability of object detection, these methods frequently suffer from high computational demands and reduced efficacy in detecting small objects and complex environments. These limitations have promoted the development of subsequent one-stage detectors.

Compared with two-stage detectors, single-stage detectors treat detection as a regression problem without the need for additional candidate region extraction. The detection system predicts object categories and locations directly from features extracted by a convolutional neural network and outputs detection results, thereby meeting real-time processing requirements. Redmon et al. [19] proposed the single-stage detection framework YOLO, which employs a single convolutional neural network and uses the whole image as the training input, producing bounding box and class probability predictions in a single pass. Since then, the YOLO series of object detection algorithms has experienced numerous iterations and enhancements, including the adoption of various backbone networks, the implementation of anchor mechanisms, multi-scale training strategies, Batch Normalization (BN) [27], FPN [28], Spatial Pyramid Pooling (SPP), Pyramid Attention Networks (PAN), the GIOU loss [29], focal mechanisms, and anchor-free detection [30], among other techniques. These advances have substantially improved both detection speed and accuracy and have driven the trend toward lightweight and efficient models. Meanwhile, Liu et al. [18] proposed the SSD algorithm to address shortcomings in object localization within the YOLO framework.

Although deep learning methods utilize convolutional neural networks to automatically learn multi-level features and thus overcome the limitations of traditional approaches that rely on hand-crafted features, they still face challenges when applied to large-scale, complex, and variable remote sensing imagery, such as small-scale target detection, target blur, and background interference.

### 2.2. Multiscale Feature Fusion

The drastic scale divergence in remote sensing imagery demands detectors with superior multi-scale representation capabilities. FPN [20] established a baseline by constructing a top-down pathway to propagate semantic information. To further enhance localization, Path Aggregation Network (PANet) [31] introduced an additional bottom-up path, while the Bidirectional Feature Pyramid Network (BiFPN) [32] implemented weighted bi-directional fusion to balance feature importance.

Recent years have witnessed the emergence of numerous advanced feature fusion architectures in diverse domains. YOLOv7 [33] proposed the E-ELAN architecture to optimize gradient path length, enabling deeper networks to learn effectively. Gold-YOLO [34] introduced a Gather-and-Distribute (GD) mechanism, replacing conventional FPNs with a global information fusion module to capture long-range dependencies. Jiang et al. [21] proposed the BDFPN architecture, which broadens the scope of the feature pyramid and leverages skip connections to effectively aggregate features across different scales. In this study, we propose a novel Cross-Channel Feature Fusion Network (MSFENet). By fully exploiting feature interactions across different channels, MSFENet addresses the limitations of previous methods regarding inter-channel dependency. Consequently, it promotes efficient multi-scale feature fusion and significantly enhances the capability to capture local detailed features.

### 2.3. Attention Mechanisms

Over recent years, scholars have applied the attention mechanism to remote sensing image target detection. The aim is to enhance the model’s ability to capture global context information through dynamic weight allocation, thereby optimizing the accuracy of feature representation for multi-scale targets in complex scenes. This mechanism adaptively focuses on key regions by calculating the correlations between elements in the feature map, effectively suppressing background interference and improving the efficiency of extracting discriminative features in remote sensing images with dense targets, variable scales, and complex backgrounds. For example, Woo et al. [35] proposed the convolutional block attention module (CBAM), which achieves feature refinement in the convolutional block through dual attention mechanisms of channel and space. In addition, the end-to-end target detection framework based on the Transformer network [36] employs a Transformer encoder and self-attention mechanisms to extract global contextual information for object detection and object relationship modeling. For instance, Carion et al. [37] proposed the DETR target detection framework based on the Transformer. This method abandons the redundant operations in the CNN target detection framework, treats target detection as a set prediction problem, and realizes end-to-end detection through the Transformer encoder–decoder structure, effectively improving the detection efficiency and reducing the complexity and computational burden of the model.

Currently, an increasing number of researchers have begun to widely employ the cross-attention mechanism. Its powerful feature interaction and screening capabilities can offer effective solutions to numerous challenges in remote sensing target detection. For example, Wang et al. [38] proposed a long-distance cross-attention module (LDCAM) to capture the dependencies between remote elements across queries and support each feature extraction layer. This module facilitates the exchange of contextual information between images, thereby achieving a more comprehensive feature representation. Lu et al. [39] proposed a semantic-guided cross-attention network (SCANet), which utilizes high-level semantic information to guide low-level spatial details through the attention mechanism. Nevertheless, these methods still treat channel and spatial features largely in a decoupled manner. In contrast, our channel-spatial cross-attention (CSCA) mechanism explicitly models bidirectional interactions between the two dimensions through a dedicated cross-attention fusion module (CAFM). Integrated within a lightweight multi-scale feature network, CSCA enhances the discriminative representation of small and multi-scale objects more efficiently, maintaining lower computational overhead than existing cross-attention designs.

## 3. Method

**Figure 2:**
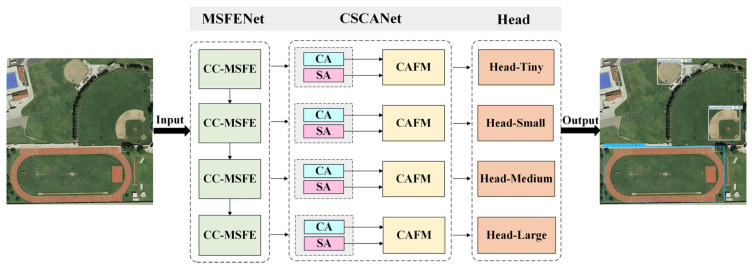
Overall framework diagram of the model.

As shown in Figure 2, we designed an object detection method based on multi-scale feature fusion and cross-attention feature enhancement for remote sensing images. This method consists of three parts: Multi-Scale Feature Extraction Network (MSFENet), Channel-Spatial Cross-Attention Mechanism Network (CSCANet), and Target Detectors. First, MSFENet is mainly composed of four cross-channel multi-scale feature extraction (CC-MSFE) modules, which process the input original image sequentially. The CC-MSFE module achieves complementary advantages among the features of different channels by cross-fusing multiple convolutional channels. It boosts the global perceptual capability of multi-scale target feature information and can efficiently capture local detailed features. Second, the four different feature maps generated by MSFENet are fed into the corresponding four branches in CSCANet, respectively. CSCANet introduces novel channel attention (CA) and spatial attention (SA), and the two attention mechanisms are deeply cross-fused by constructing the cross-attention fusion module (CAFM) to achieve dynamic interaction and joint optimization in the channel and spatial dimensions. Finally, four scale detection heads are used to locate and detect the targets at varying scales.

### 3.1. Cross-Channel Multi-Scale Feature Extraction

**Figure 3:**
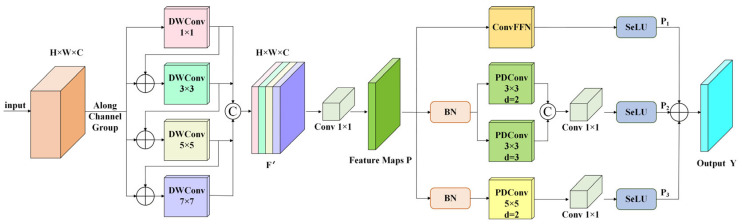
Structure of CC-MSFE module.

The CC-MSFE module is a feature extraction unit designed for multi-scale target detection tasks. Its primary role is to extract and integrate cross-channel features across varying scales, facilitating efficient feature representation of targets of various sizes (especially small targets) in complex scenarios, while balancing the accuracy of feature extraction and computational efficiency. The framework structure is shown in Figure 3.

First, the CC-MSFE module divides the input feature map $X\in R^{H\times W\times C}$ into four groups along the channel dimension, as follows:

(1) $$X_{1},X_{2},X_{3},X_{4}=Split\left(X\right),X_{i}\in R^{H\times W\times \frac{C}{4}},i=1,2,3,4$$

where *H* represents the height of the feature map, *W* represents the width of the feature map, and C represents the number of channels. When the channel number C is not divisible by 4, padding is applied by adding zero-valued channels so that the total number of channels becomes divisible by 4. $C^{\prime }=4\times \lceil C/4\rceil$, [·] represents the rounding operation.

Then, each group performs parallel depthwise separable convolutions (DWConv). Subsequently, the obtained feature maps are concatenated with the other group of feature maps to enhance the interaction of feature information among different channels, thereby achieving multi-scale feature extraction.

In the first group $X_{1}$, we employed a 1 × 1 depthwise separable convolution, and the formula is as follows:

(2) $$F_{1}=DWConv_{1\times 1}$$

Then, the feature map $F_{1}$ is concatenated with the second group $X_{2}$, and the concatenated features are processed using a 3 × 3 depthwise separable convolution to obtain a new feature map $F_{2}$. By repeating the above operations, four groups of different feature maps can be obtained. These feature maps are concatenated, the channel dimension is compressed and information is fused through a 1 × 1 convolution to generate Feature Maps P. The formula is as follows:

(3) $$F_{i}=DWConv_{k_{i}\times k_{i}}\left(X_{i}+F_{i-1}\right),i=2,3,4,k_{2}=3,k_{3}=5,k_{4}=7$$

(4) $$F^{\prime }=Concat\left(F_{1},F_{2},F_{3},F_{4}\right),F^{\prime }\in R^{H\times W\times C}$$

(5) $$P=Conv_{1\times 1}\left(F^{\prime }\right)$$

Feature Maps P are processed through three parallel branches. In the first branch, the Convolutional Feed-Forward Network (ConvFFN) is utilized for information interaction and nonlinear transformation among channels, followed by the use of the SeLU activation function. The formula is as follows:

(6) $$P_{1}=SeLU\left(ConvFFN\left(P\right)\right)$$

In the second branch, batch normalization (BN) is first applied. Preliminary experiments have shown that performing BN before convolution operations can accelerate the convergence speed of the model. Next, parallel dilated convolution (PDConv) using 3 × 3 kernels with dilation rates d = 2 and d = 3 captures spatial features at different receptive fields concurrently. The two convolution outputs are concatenated, fused and reduced in dimension by a 1 × 1 convolution, and the result is passed through the SeLU activation. The formulation is as follows:

(7) $$P^{\prime }=BN\left(P\right)$$

(8) $$Q_{1}=Concat\left(PDConv_{3\times 3,d=2}\left(P^{\prime }\right),PDConv_{3\times 3,d=3}\left(P^{\prime }\right)\right)$$

(9) $$P_{2}=SeLU\left(Conv_{1\times 1}\left(Q_{1}\right)\right)$$

The third branch is similar to the second one. The convolution operation is changed to parallel dilated convolution (PDConv) with a convolution kernel of 5 × 5 (dilation rate d = 2), and the formula is as follows:

(10) $$Q_{2}=PDConv_{5\times 5,d=2}\left(P^{\prime }\right)$$

(11) $$P_{3}=SeLU\left(Conv_{1\times 1}\left(Q_{2}\right)\right)$$

Finally, the output feature maps of the three branches are concatenated to obtain the output feature map Y.

(12) $$Y=P_{1}+P_{2}+P_{3}$$

### 3.2. Channel-Spatial Cross-Attention Mechanism

We designed a novel channel-spatial cross-attention mechanism, which aims to further expand the receptive field of the model, enhance the representational ability of target features, and improve the adaptability to targets of different scales. This mechanism mainly consists of three major components: channel attention, spatial attention, and a cross-attention fusion module. We optimized channel and spatial attention and devised the CAFM to cross-integrate them, thereby precisely focusing on key features and improving the model’s robustness and discriminative power.

#### 3.2.1. Channel Attention

**Figure 4:**
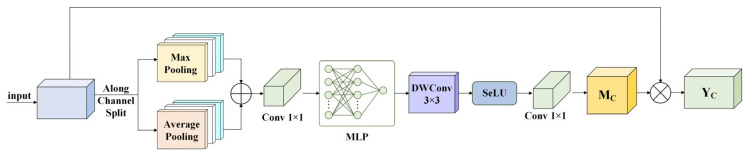
Structure of CA module.

The CA module is an efficient enhancement of the classic channel attention mechanism. By introducing multi-branch pooling, non-linear transformation, and depthwise convolution, it dynamically generates channel weights to achieve more precise calibration of key features, as shown in Figure 4.

First, the feature map $U\in R^{H\times W\times C}$ is split along the channel dimension into $U_{1}$ and $U_{2}$. The two branches perform Max Pooling and Average Pooling, respectively, and then the results of the branches are added together. The added feature vectors first pass through a 1 × 1 convolution that performs a linear transformation and reduces dimensionality, lowering the complexity of subsequent computations. Next, a multilayer perceptron (MLP) module and a 3 × 3 depthwise separable convolution ($DWConv_{3\times 3}$) are used to further enhance local context awareness and the ability to capture key information. After that, the Scaled Exponential Linear Unit (SeLU) activation function is adopted to ensure the non-linear fitting ability of attention weights, followed by a 1 × 1 convolution to produce the channel attention weights $M_{c}$. Finally, the channel attention weights $M_{c}$ are multiplied by the original input feature map U to amplify the contributions of critical channels and suppress the responses of unimportant or noisy channels. The calibrated output feature map $Y_{c}$ is finally obtained.

(13) $$V=MLP\left(Conv_{1\times 1}\left(MaxPool\left(U_{1}\right)+AvgPool\left(U_{2}\right)\right)\right)$$

(14) $$M_{c}=Conv_{1\times 1}\left(SeLU\left(DWConv_{3\times 3}\left(V\right)\right)\right)$$

(15) $$Y_{c}=M_{c}⊗U$$

#### 3.2.2. Spatial Attention

**Figure 5:**
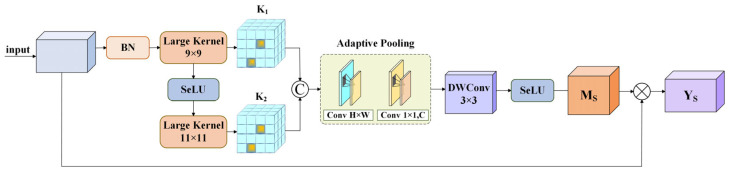
Structure of SA module.

The SA module utilizes large-kernel convolution and adaptive pooling strategies to capture wide-range spatial context, enabling dynamic focusing on the key spatial regions of the input feature map. This strengthens the model’s capability to understand spatial relationships and recognize important spatial positions, as shown in Figure 5.

First, the input feature map $U\in R^{H\times W\times C}$ undergoes batch normalization (BN), followed by a large-kernel convolution with a 9 × 9 kernel. One branch yields $K_{1}$, while the other branch applies the SeLU activation function first and then a second large-kernel convolution with an 11 × 11 kernel to produce $K_{2}$. Next, the feature map obtained by concatenating $K_{1}$ and $K_{2}$ passes through an adaptive pooling module, which captures feature information at different scales by dynamically adjusting the window size. In addition, a 3 × 3 depthwise separable convolution further supplements fine-detail information and employs the SeLU activation. Finally, the resulting spatial attention weight $M_{s}$ is multiplied with the original input feature map U via matrix multiplication to obtain the output feature map $Y_{s}$.

(16) $$K_{1}=Conv_{LargeK}\left(BN\left(U\right)\right)$$

(17) $$K_{2}=Conv_{LargeK}\left(SeLU\left(K_{1}\right)\right)$$

(18) $$M_{s}=SeLU\left(DWConv_{3\times 3}\left(AP\left(Concat\left(K_{1}+K_{2}\right)\right)\right)\right)$$

(19) $$Y_{s}=M_{s}⊗U$$

#### 3.2.3. Cross-Attention Fusion Module

**Figure 6:**
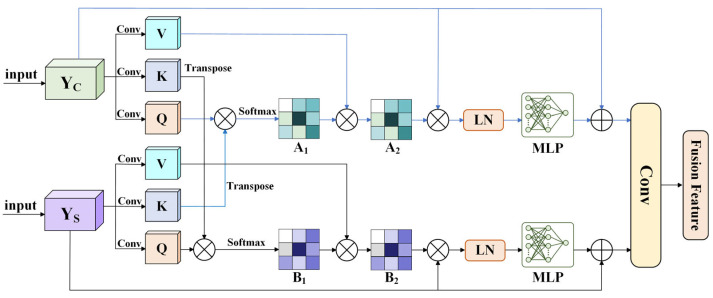
Structure of CAFM module.

CAFM achieves deep interaction and integration between channel-attention and spatial-attention features. The two are regulated bidirectionally, which effectively fuses channel and spatial information and substantially enhances the model’s capacity to identify targets in complicated backgrounds, as shown in Figure 6.

The module first receives the output feature $Y_{c}$ from the CA module and the output feature $Y_{s}$ from the SA module. It then performs collaborative computation via two parallel cross-attention branches. Each input feature is projected by an independent Conv-layer to produce three vectors: Query (Q), Key (K) and Value (V).

(20) $$Q_{c},K_{c},V_{c}=Conv\left(Y_{c}\right)$$

(21) $$Q_{s},K_{s},V_{s}=Conv\left(Y_{s}\right)$$

Branch 1 is spatial feature-guided channel enhancement, which enables the key position information in the space to filter and enhance the channel features most relevant to it. The computation is given by the following formula:

(22) $$A_{1}=softmax\left(\frac{Q_{c}K_{s}^{T}}{\sqrt{d_{k}}}\right)$$

(23) $$A_{2}=V_{c}⊗A_{1}$$

where $d_{k}$ denotes the dimensionality of the key vector $K_{s}$, and the term $\sqrt{d_{k}}$ serves as a scaling factor to ensure that the input to the softmax function remains within an appropriate numerical range.

Branch 2 is channel feature-guided spatial enhancement, enabling important channel information to guide the focus on key spatial regions. The corresponding formula is expressed as follows:

(24) $$B_{1}=softmax\left(\frac{Q_{s}K_{c}^{T}}{\sqrt{d_{k}}}\right)$$

(25) $$B_{2}=V_{s}⊗B_{1}$$

where $d_{k}$ denotes the dimensionality of the key vector $K_{c}$.

The weight matrices output by the two pathways are each multiplied by the original features, undergo feature transformation via layer normalization (LN) and a multi-layer perceptron (MLP), and are finally residual-connected to the original input features and fused through a convolutional layer to produce the final feature representation.

## 4. Experiments

### 4.1. Datasets

**Figure 7:**
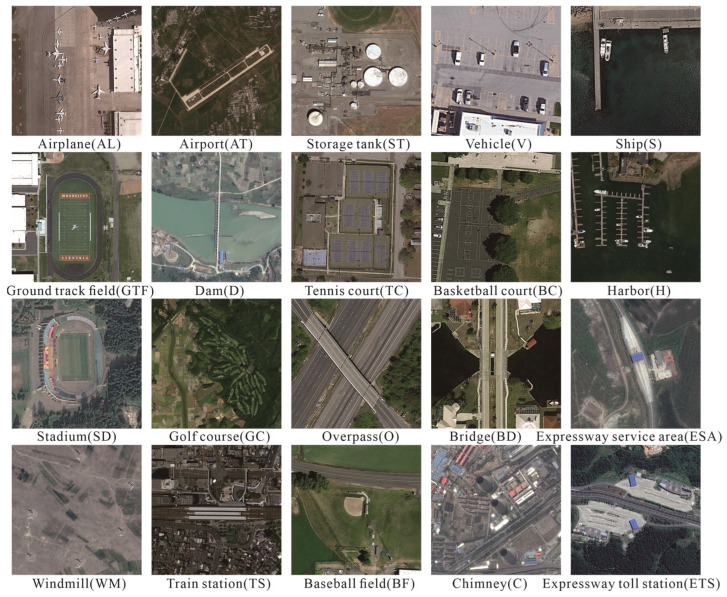
Examples of 20 classes in DIOR dataset.

**Figure 8:**
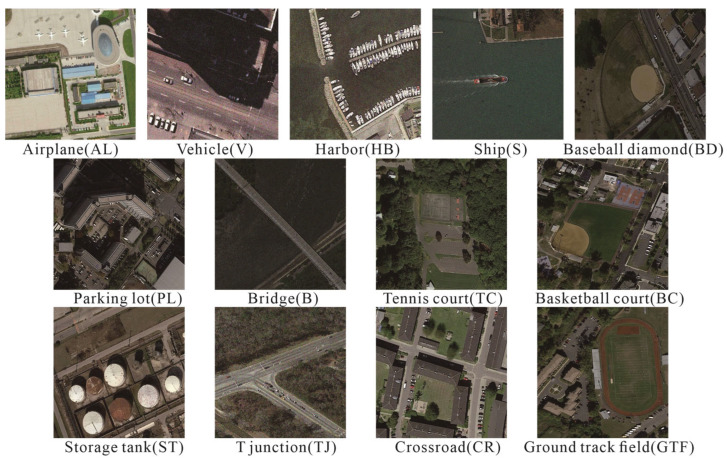
Examples of 13 classes in HRRSD dataset.

**Figure 9:**
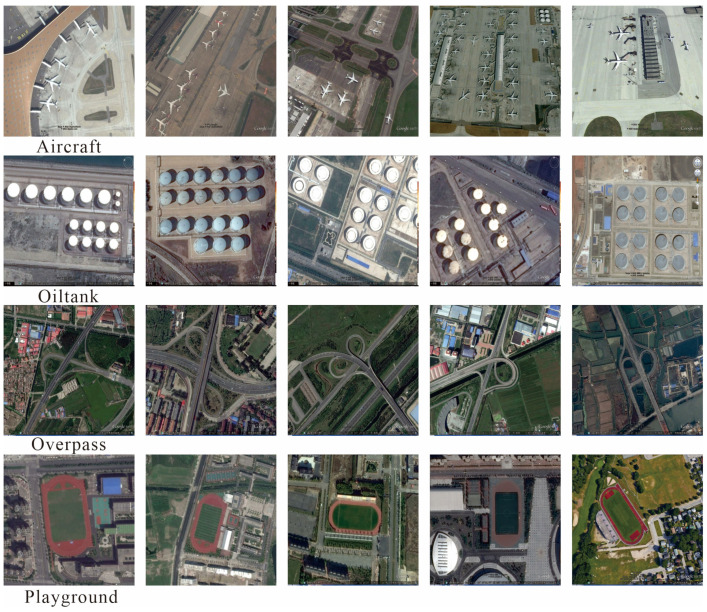
Examples of 4 classes in RSOD dataset.

We selected three publicly available and challenging datasets: detection in optical remote sensing images (DIOR), high-resolution remote sensing detection (HRRSD) and remote sensing object detection (RSOD). Since the number of images contained in different categories varies among the three datasets, there is a class imbalance phenomenon in all of them. The specific introduction is as follows:

(1) The detection in optical remote sensing images (DIOR) dataset is a large-scale public benchmark dataset for remote sensing image object detection [11]. This dataset contains 23,463 images and 192,472 instances, covering 20 common ground object categories, such as airplanes, airports, ships, vehicles, bridges, etc., as shown in Figure 7. The image size is uniformly 800 × 800 pixels, with a spatial resolution ranging from 0.5 m to 30 m. The images are collected from more than 80 countries and regions around the world, including different seasons, lighting conditions, and angles. In the DIOR dataset, we randomly selected 8207 images as the training set and 3518 images as the validation set from 11,725 images in a 7:3 ratio, with the remaining 11,738 images used as the test set.

(2) The high-resolution remote sensing detection (HRRSD) dataset is a large-scale remote sensing image dataset for multi-class object detection tasks [40]. This dataset includes 13 common categories, such as airplanes, baseball fields, bridges, and ships, with a total of over 20,000 color images, as shown in Figure 8. The images are mainly sourced from Google Earth, with a spatial resolution ranging from 0.15 m to 1.2 m. The large data scale and diverse covered scenarios help improve the generalization ability of the model. The dataset is partitioned into a training set, a validation set, and a test set through algorithm optimization. It has large intra-class differences and high inter-class similarities, posing certain challenges.

(3) The remote sensing object detection (RSOD) dataset is an open remote sensing image object detection dataset [41]. This dataset includes a total of 976 remote sensing images, containing 6950 high-quality annotated instances, covering four typical target categories: aircraft, oil tanks, overpasses, and playgrounds. The spatial resolution ranges from 0.3 m to 3 m, as shown in Figure 9. This dataset covers a variety of scenes and angles, which helps algorithms adapt to object detection in different environments and can effectively verify the model’s detection performance for small-scale targets in complex scenes. We randomly divided the images in each category into a training set and a validation set at a ratio of 7:3, with 651 images in the training set and 285 images in the validation set.

### 4.2. Experimental Setup and Evaluation Metrics

#### 4.2.1. Experimental Setup

The experimental environment and training parameters are summarized in Table 1. All experiments were conducted on a high-performance computing platform equipped with a Xeon^®^ Platinum 8470Q CPU and an NVIDIA RTX 5090 GPU with 32 GB memory, running Ubuntu 22.04. The model was implemented using PyTorch 2.1.2 with CUDA 12.1. All input images were uniformly resized to 800 × 800 pixels.

Given the complexity and domain specificity of remote sensing datasets, no pre-trained weights were employed; instead, the network was trained, validated, and tested entirely from scratch on the target datasets. The training process adopted a cross-validation strategy to ensure robustness. An initial learning rate of 0.0001 was used with a momentum coefficient of 0.937. To stabilize optimization, a linear warm-up strategy was applied at the early training stage, followed by a cosine annealing schedule to gradually decrease the learning rate and facilitate better convergence. Mosaic data augmentation was introduced during training to enhance data diversity and improve the model’s generalization ability under complex and cluttered backgrounds. Additionally, a weight decay of 0.0005 was applied as a regularization term to mitigate overfitting. The batch size was set to 16, and the model was trained for a total of 300 epochs.

It should be noted that for some compared methods, the official implementations are not publicly available. Consequently, their model parameters and FLOPs cannot be re-evaluated under the exact same hardware environment and are therefore directly taken from the original papers. Except for hardware-related differences, the software configuration, experimental settings, and evaluation pipeline are kept consistent with those described in the corresponding literature. Under this condition, the reported results still provide a reliable reference for comparing detection performance across different methods.

#### 4.2.2. Evaluation Metrics

In this study, we principally employ Average Precision (AP) and mean Average Precision (mAP) as the core metrics to quantify and evaluate the model. *AP* is the area under the precision-recall (P-R) curve, which comprehensively reflects the model’s performance across different recall levels. mAP is the arithmetic mean of the *AP* values across all classes and is used to mitigate evaluation bias arising from class imbalance. The computation is given by the following formulas:

(26) $$AP=\int_{0}^{1}P\left(R\right)dt$$

(27) $$mAP=\frac{1}{N}\sum_{n=1}^{N}AP_{n}$$

where *P* represents the proportion of the number of correctly detected results to the total number of all detection results, *R* represents the proportion of the number of correctly detected targets to the total number of actual targets, and *N* represents the total number of target categories in the dataset.

mAP50 is the average *AP* of all categories under the condition of IoU = 0.5. It is a key indicator that balances practicality and efficiency in target detection, and it balances the positioning accuracy and the model’s fault tolerance ability through a moderate IoU threshold. mAP50:95 is a comprehensive indicator (with the IoU ranging from 0.5 to 0.95), which comprehensively measures the model’s classification and positioning abilities under different positioning accuracy requirements.

In addition, to account for the large-scale variation of objects in remote-sensing images, this study adopts the COCO grading evaluation system: APs (Average Precision of Small) for areas less than 32^2^ pixels, APm (Average Precision of Medium) for areas between 32^2^ and 96^2^ pixels, and APl (Average Precision of Large) for areas greater than 96^2^ pixels.

Finally, to evaluate the model’s computational efficiency, we computed model parameters to quantify storage requirements and calculated FLOPs to infer the model’s running speed. To reduce experimental randomness, we repeated each experiment ten times and report the mean of the ten results.

### 4.3. Experimental Results

#### 4.3.1. Results in the DIOR Dataset

We selected 13 mainstream object detection models for comprehensive comparative analysis on the DIOR dataset, as shown in Table 2. The comparison models mainly include single-stage detector models (RetinaNet [42]), two-stage detector models (Faster R-CNN [43] and Cascade R-CNN [44]), transformer-based models (RT-DETR [23] and ViTDet [45]), classic YOLO series models (YOLOv5 [46], YOLOv7 [33], YOLOv8 [47], YOLOX [48], YOLOv11 [49], Super-YOLO [50], and Gold-YOLO [34]), and the latest model MCFM [51].

The experimental results are shown in Table 2. In terms of overall performance, our model achieves the best results. The mAP value of our model reaches 78.1%, representing improvements of 26% and 8.2% compared to the classic two-stage detectors, Faster R-CNN and Cascade R-CNN, respectively. Compared with the single-stage detector RetinaNet, the mAP increases by 10.3%. In comparison with the transformer-based RT-DETR, the mAP increases by 4.9%. When compared with YOLOv5 and YOLOv11 in the YOLO series, the improvements are 4.3% and 0.7%, respectively. Moreover, our model surpasses the recently proposed advanced model MCFM (77.6%) by 0.5%, which demonstrates the advancement and effectiveness of our model in remote sensing object detection tasks. Furthermore, with respect to detection performance on individual categories, our model maintains advantages over competing methods. For example, it achieves the best performance in ship (S, 92.1%), tennis court (TC, 65.3%), and overpass (O, 65.3%) categories, while also ranking among the top performers in airplane (AL, 83.2%), chimney (C, 77.5%), dam (D, 66.8%), and storage tank (ST, 86.2%).

The experimental results presented above demonstrate that the advanced architecture model we designed, which combines multi-scale perception and channel cross, has strong generalization ability and can stably handle diverse ground object targets in remote sensing scenarios. By cross-fusing channel attention and spatial attention, the model’s adaptability to complex scenes is further strengthened. In terms of computational efficiency, compared with other models, the number of parameters and computational complexity of our model are at a relatively low level, achieving model lightweight while improving computational efficiency.

#### 4.3.2. Results in the HRRSD Dataset

The experimental results are shown in Table 3. Compared with the other 11 object detection models, the mAP of our model on the HRRSD dataset reaches 90.6%, which is 5.8% higher than that of the single-stage detector CornerNet [52], 11.1% higher than that of the two-stage detector Faster R-CNN, 16.4% higher than that of the vision transformer-based ViTDet, and 0.6% and 1.4% higher than those of the classic Gold-YOLO and YOLOv11 algorithms, respectively. In addition, the proposed algorithm performs excellently in most specific detection categories of the HRRSD dataset. The model ranks first in terms of accuracy in a total of 7 categories, including airplanes (AL, 99.1%), baseball fields (BC, 77.1%), bridges (BD, 87.7%), crossroads (CR, 95.1%), harbors (HB, 95.9%), parking lots (PL, 67.7%), and storage tanks (ST, 97.2%). The model leads performance in challenging categories such as parking lots and bridges, demonstrating the strong capability of its cross-attention mechanism to detect complex small targets.

Compared with Super-YOLO and YOLOv11, which have similar computational costs, our model not only shows certain advantages in mAP but also significantly improves the detection performance for specific categories. This further demonstrates that the model has good stability and robustness, achieving a balance between efficiency and performance. It also confirms the enhancing effect of the multi-scale cross-channel fusion mechanism and the attention cross mechanism on the detection performance.

#### 4.3.3. Results in the RSOD Dataset

As shown in Table 4, this table presents the comparison results of our model with 8 other remote sensing object detection models on the RSOD dataset. Our model achieved 96.5% mAP. It substantially surpasses two-stage detectors such as Faster R-CNN (69.6%) and single-stage detectors such as RetinaNet (80.4%), and it also outperforms members of the YOLO series, including YOLOv7 (92.8%), YOLOv8 (94.4%), and YOLOv10 (89.7%) [54]. In particular, our model outperforms the latest GAM (95.9%) [55] based on the global attention mechanism by 0.6%, indicating that our model has high accuracy in detecting objects. In the aircraft and oiltank categories, our model achieves detection accuracies of 98.1% and 97.5%, respectively, ranking first in both. In the Overpass category, which features complex structures and substantial background interference, our model achieved 93.3% accuracy, 2.5% higher than the second-ranked GAM (90.8%). This result demonstrates the model’s precise recognition of targets with complex structures.

Further analysis indicates that, compared with the DIOR and HRRSD datasets, the RSOD dataset exhibits pronounced class imbalance. For example, there are 446 images of aircraft but only 165 images of oiltank, which may lead to insufficient feature learning for minority classes. To address such issues, the YOLO series models mainly adopt loss function design, model structure optimization, and advanced training strategies. However, these approaches have not fully resolved the problem of inadequate feature extraction and can sometimes do so at the expense of overall computational efficiency. In contrast, the model we proposed mainly concentrates on critical information within the image through the CSCA mechanism, enhancing its feature learning and discrimination ability. This helps to compensate for the weak feature representation ability caused by the small amount of data and achieves good results in the evaluation metric mAP.

Compared with YOLOv10, although the computational parameters and complexity of our model slightly increase, the detection accuracy is significantly improved. Compared with YOLOv5 with similar computational cost, our model shows obvious improvements in detection performance both overall and for specific categories. The results confirm the superior ability of the proposed algorithm for the detection accuracy of multi-scale objects and computational cost.

**Figure 10:**
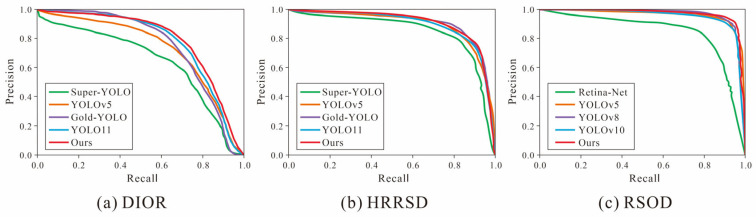
P-R curves of each method on three datasets. (**a**) DIOR. (**b**) HRRSD. (**c**) RSOD.

Finally, on the three remote sensing datasets of DIOR, HRRSD, and RSOD, the P-R curves of our model are compared with those of multiple current mainstream object detection methods, as shown in Figure 10. The P-R curve reflects the relationship between the precision and recall of the model under different confidence thresholds. The area under the curve corresponds to the mean average precision (mAP). A larger area and a curve nearer the upper right indicate better overall detection performance of the model.

On the DIOR dataset, the P-R curve of our algorithm significantly approaches the upper-right corner, and the area enclosed by the curve is larger than that of other comparison methods, indicating that our model has higher detection accuracy and stronger comprehensive detection ability. On the HRRSD and RSOD datasets, the P-R curve of our model also shows a stable and leading curve shape, suggesting that our model has good adaptability and generalization ability on datasets of different scales.

### 4.4. Ablation Experiment and Analysis

To verify the effectiveness and role of each module in the overall detection performance, we designed systematic ablation experiments on the DIOR, HRRSD, and RSOD datasets. The experimental details are presented below.

(1) Effects of CC-MSFE: The proposed CC-MSFE module utilizes cross-channel fusion and depthwise separable convolutions to extract multi-scale features, improving target perception across sizes while retaining a lightweight architecture. As shown in Table 5, ablation results confirm the necessity of both components across all three datasets. On the DIOR dataset, replacing the depthwise separable convolution with ordinary convolution and canceling the cross-channel fusion mechanism leads to a 6% decrease in mAP50 and a 3.6% decrease in mAP50:95, where the cross-channel design plays a major role. In addition, APs decreases by 10.9%, APm decreases by 10%, and APl decreases by 0.4%, indicating that the design of depthwise separability and cross-channel mainly enhances the detection precision for small and medium targets. This trend is consistent on the HRRSD and RSOD datasets. Notably, in the enhanced model, the parameter count (Para) only increases from 19.0 M to 19.5 M, and FLOPs rise from 74.5 G to 75.2 G, demonstrating a significant performance improvement with almost no additional computational cost.

(2) Effects of CA and SA: The main functions of the CA and SA modules we designed are to enhance the model’s ability to capture important information. The experimental results demonstrate that introducing either CA or SA individually leads to consistent performance improvements. Specifically, CA mainly enhances the semantic discriminability along the channel dimension, whereas SA significantly strengthens the model’s capability to capture spatial location information of targets, exhibiting particularly notable gains in small-object detection tasks. Under the combined enhancement from the two modules, the model achieved significant improvements in mAP50 and mAP50:95 across all three datasets. Notably, the CA module contributed to APs increases of 2.9%, 2.0%, and 1.4% on the DIOR, HRRSD, and RSOD datasets, respectively, while the SA module improved APs by 4.0%, 1.8%, and 1.6% on the same datasets. These findings indicate that CA and SA play complementary roles in optical remote sensing object detection.

(3) Effects of CAFM: We designed the CAFM to better cross-fuse channel attention and spatial attention, enhancing the model’s comprehensive understanding of multi-source information in complex backgrounds. As shown in Table 6, the integration of the CAFM significantly improved model performance, increasing mAP50 by 3.8%, 5.1%, and 4.9% on the DIOR, HRRSD, and RSOD datasets, respectively. The most pronounced gains were observed on DIOR, with APs, APm, and APL increasing by 5.6%, 9%, and 1.9%, which clearly validates the role of CAFM in optimizing object detection performance. While reducing the computational complexity, it achieved a significant improvement in detection accuracy and robustness.

To further verify the effectiveness and practical value of the proposed CSCA attention mechanism, we conducted additional comparative experiments. As shown in Table 7, on the DIOR dataset, CSCA achieves an mAP50:95 of 67.0%, which is noticeably higher than that of SK [57] (65.3%) and CA [58] (65.9%). Compared with Wavelet Attention [59], CSCA improves the small-object detection accuracy by 5.1%, indicating a stronger capability to recognize small targets under complex backgrounds. On the HRRSD dataset, the superiority of CSCA is further validated, where it attains an mAP50 of 92.2%, outperforming SK, CA, and Wavelet Attention by 1.0%, 0.4%, and 0.2%, respectively. On the RSOD dataset, CSCA likewise delivers the best overall performance. Notably, CSCA incurs a lower parameter efficiency (19.5 M Params, 75.2 G FLOPs) than SK (28.2 M Params, 85.3 G FLOPs) and Wavelet Attention (20.3 M Params, 76.1 G FLOPs), demonstrating a favorable trade-off between detection accuracy and computational complexity.

### 4.5. Visualization Results

**Figure 11:**
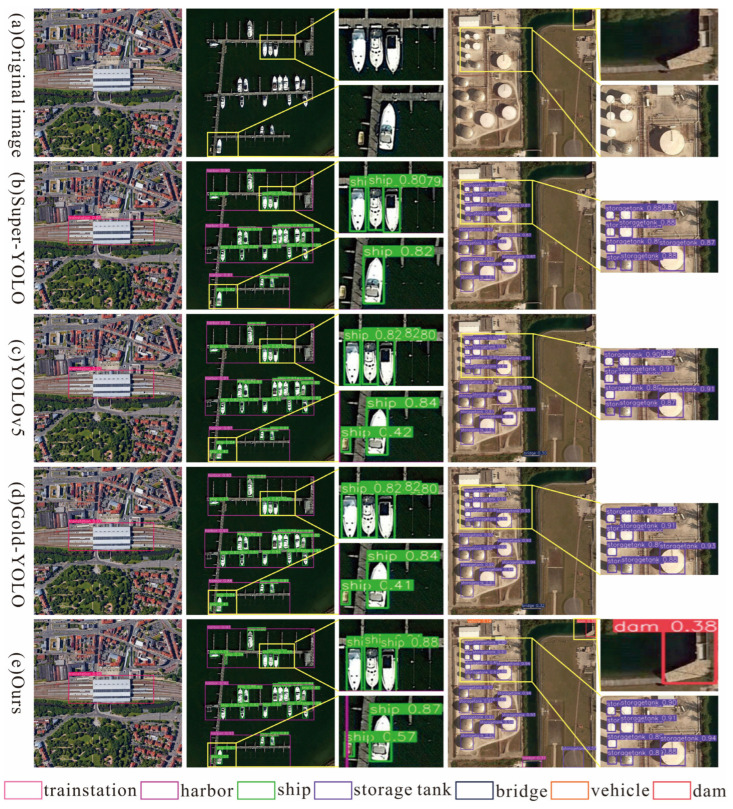
Visualization of detection results by various methods on the DIOR dataset.

**Figure 12:**
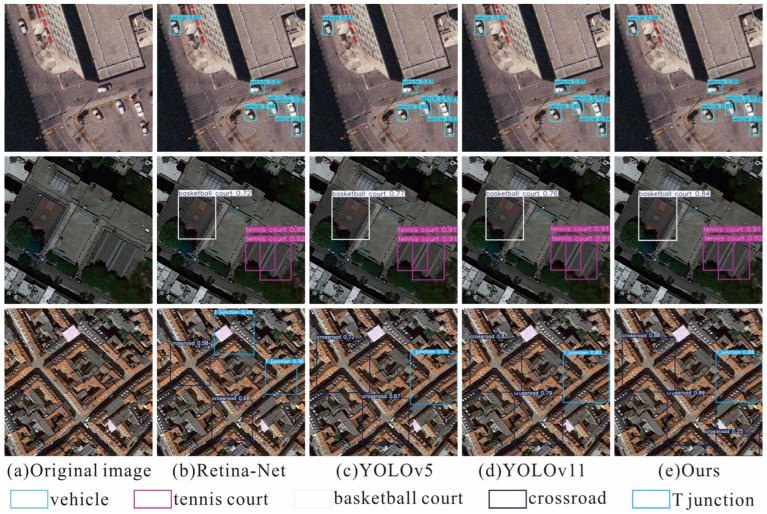
Visualization of detection results by various methods on the HRRSD dataset.

**Figure 13:**
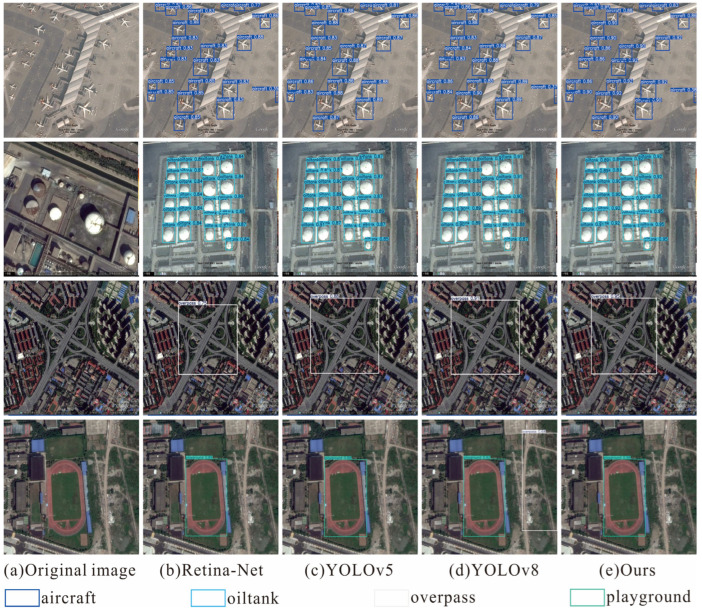
Visualization of detection results by various methods on the RSOD dataset.

**Figure 14:**
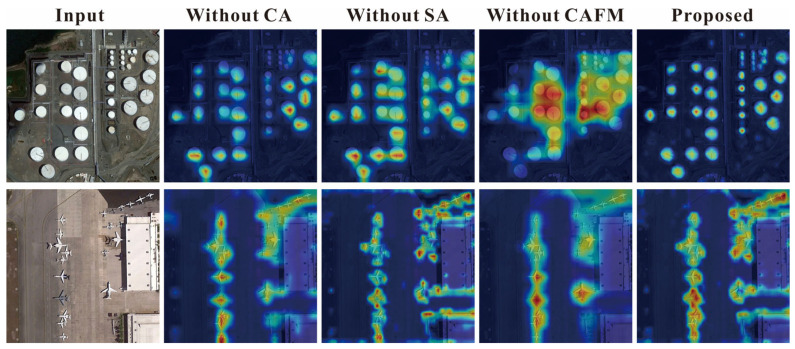
Visualization of feature maps from the ablation study on CSCANet.

In addition to quantitative comparisons, we also visualize detection results and the feature maps in Figure 11, Figure 12, Figure 13 and Figure 14 to provide an intuitive understanding of our proposed methods.

#### 4.5.1. Visualization of Detection Results

On the three datasets of DIOR, HRRSD, and RSOD, we visually present the detection results of our model and other models, as shown in Figure 11, Figure 12 and Figure 13.

In the DIOR dataset, we selected three types of remote sensing images with different detection categories for comparison with other models. As shown in Figure 11, our model achieves the best detection results and exhibits good robustness. For instance, the first row of images shows the detection results of train stations in a complex urban background. Our model can effectively overcome background interference, accurately identify and locate targets, and achieve the highest detection accuracy. The second row of images presents the detection results of large-scale targets (harbors) and small-scale targets (ships). The proposed model demonstrates robust multi-scale adaptability, delivering balanced accuracy in localizing and classifying targets of varying sizes. The third row of images displays detection results for storage tanks with high inter-class similarity. Through the synergistic optimization of the multi-scale feature extraction module and attention mechanism module, the model can precisely identify subtle feature differences.

In the HRRSD dataset, we also selected remote sensing images from three different scenarios for verification, as shown in Figure 12. The first scenario involves small-target detection in complex backgrounds. For example, the images in the first row illustrate the detection of small vehicles in street scenes. The second scenario focuses on target detection under occlusion. Specifically, the images in the second row show the detection of basketball courts and tennis courts partially occluded by vegetation. The third scenario addresses the detection of objects with similar features. As illustrated in the third row, the model can accurately detect crossroads and T-junctions in similar traffic road scenes. The results indicate that our model exhibits good robustness and generalization ability in the above scenarios.

In the RSOD dataset, we visually compare the remote sensing images of four typical targets with other detection models, as shown in Figure 13. The detection results show that our model exhibits stable and excellent detection performance in different scenarios. Specifically, for densely parked airplanes (first row) and densely arranged oil tank groups (second row), our model maintains high positioning accuracy and recall rate under complex spatial distributions, effectively verifying its excellent feature recognition and anti-occlusion capabilities in high-density small target detection. For large-scale targets such as overpasses with complex structures (third row) and sports fields (fourth row), our model can still accurately capture the overall contours of the targets, demonstrating its strong perception and discrimination abilities for multi-scale targets.

#### 4.5.2. Visualization of Feature Maps

As shown in Figure 14, the feature maps from the ablation study on CSCANet visually demonstrate the functions and synergistic effects of its constituent modules. Removing either the channel attention (CA) or spatial attention (SA) module noticeably weakens the representation of small-object features and leads to missed detections. The degradation becomes more pronounced when the cross-attention fusion module (CAFM) is removed, where boundary cues are blurred and missed detections are more frequent in densely populated small-object scenes. In contrast, the complete model exhibits stronger discriminative capability for target features and delineates object locations and boundaries more accurately, highlighting the complementary and synergistic roles of CA, SA, and CAFM in improving feature representation and localization precision.

**Figure 15:**
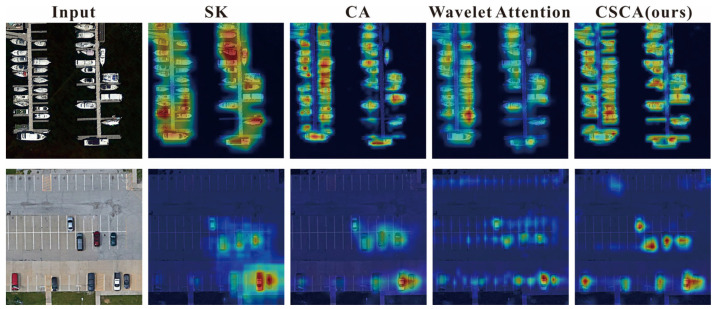
Visual comparison of our proposed attention mechanism with other attention mechanisms.

Figure 15 shows the effectiveness of the proposed CSCA attention mechanism in comparison with other attention mechanisms, including SK, CA, and Wavelet Attention. SK and CA fail to clearly distinguish targets from surrounding background structures, resulting in diffuse and incomplete responses. Compared with other attention mechanisms, CSCA can better highlight crucial regions and preserve finer-grained features, particularly demonstrating superior performance in dense small-object detection tasks (e.g., ships and vehicles), which indicates that CSCA has a stronger capability in representing and discriminating small targets under complex backgrounds.

## 5. Conclusions

This study proposes a multi-scale object detection network with integrated spatial-channel collaborative attention, comprising four key modules: cross-channel multi-scale feature extraction (CC-MSFE), channel attention (CA), spatial attention (SA), and channel-spatial cross-attention fusion mechanism (CAFM). To address the issue of insufficient extraction of multi-scale target features, we mainly enhance the feature perception ability of multi-scale targets (especially small targets) through the cross-channel multi-scale feature extraction module, achieving complementarity among features of different channels. Regarding the problem of insufficient feature fusion between space and channels, we innovatively design the channel-spatial cross-attention mechanism. By deeply fusing spatial and channel attention, the context perception ability is significantly improved, and precise focusing on key information is achieved. Finally, we experimentally validated the method on three challenging public remote-sensing datasets: DIOR, HRRSD, and RSOD. The results show that the mAP values of our model on the three datasets reach 78.1%, 90.6%, and 96.5%, respectively, significantly outperforming comparative models such as Faster R-CNN, RetinaNet, and YOLOv11. Notably, the model maintains a low level in terms of the number of parameters (19.5 M) and computational complexity (75.2 G FLOPs), achieving a favorable trade-off between detection accuracy and operational efficiency.

Although the model proposed in this study has achieved good performance in multiple aspects, there are still some limitations. For instance, our model has not been tested on real-scene images. As real scenes usually contain more noise, complex backgrounds, and are affected by cloud and fog interference, the generalization ability of the model needs further verification, and the inference speed will also be affected to some extent. In the future, we will further optimize the model and validate it in real scenes.

## Figures and Tables

**Table 1:** Experimental environment parameters.

Item	Content
CPU	Xeon^®^ Platinum 8470Q
GPU	NVIDIA RTX 5090
Memory	32 GB
OS	Ubuntu 22.04
Python	3.10.8 (64-bit)
PyTorch	2.1.2
CUDA	12.1
Learning rate	0.0001
Momentum	0.937
Weight decay	0.0005
Batch	16
Input size	800 × 800

**Table 2:** Quantitative comparison of our algorithm with other models on the DIOR dataset. (The best results are highlighted in red, the second-best results are highlighted in blue, and the third-best results are highlighted in purple).

Model	AL	AT	BC	BD	BF	C	D	S	O	V	H	ETS
Faster R-CNN [43]	37.7	61.8	73.0	32.8	65.8	71.1	45.3	34.4	47.2	30.2	44.9	46.4
RetinaNet [42]	74.7	69.4	88.0	37.2	81.3	74.0	61.3	67.4	56.3	46.1	57.1	61.6
Cascade R-CNN [44]	81.6	80.3	81.3	46.2	80.2	71.9	65.8	69.4	60.4	49.5	47.8	65.3
RT-DETR [23]	75.5	81.5	86.0	46.2	82.2	75.9	63.3	88.7	60.9	54.4	61.4	73.5
ViTDet [45]	77.8	64.6	68.8	38.4	85.0	80.3	44.2	43.9	53.3	46.1	35.6	69.0
YOLOv5 [46]	80.7	71.8	90.0	50.1	83.9	78.3	54.4	90.8	60.7	60.0	57.1	79.7
YOLOv7 [33]	83.0	78.5	89.3	49.9	85.7	78.4	61.5	90.4	60.8	58.1	62.4	76.3
YOLOv8 [47]	77.5	83.0	89.1	47.3	84.7	76.7	66.5	91.8	62.5	54.7	65.0	71.6
YOLOX [48]	83.2	79.3	90.1	51.1	85.2	78.1	63.8	91.5	63.5	59.4	64.6	78.6
Super-YOLO [50]	77.6	69.9	87.5	50.3	83.5	74.5	51.7	86.9	60.6	45.8	47.3	70.1
Gold-YOLO [34]	73.9	87.2	88.2	47.3	82.6	77.3	66.1	89.6	62.9	49.3	65.4	68.3
YOLOv11 [49]	85.6	84.5	90.5	50.4	83.9	77.8	66.8	91.8	64.1	59.8	64.7	76.1
MCFM [51]	81.3	83.7	89.8	52.5	83.7	76.6	67.1	91.4	64.3	60.0	64.1	77.2
ours	83.2	81.6	90.2	51.4	85.3	77.5	66.8	92.1	65.3	58.7	65.0	78.5
**Model**	**GC**	**TC**	**TS**	**WM**	**SD**	**ST**	**ESA**	**GTF**	**mAP**	**Para (M)**	**FLOPs(G)**	
Faster R-CNN [43]	65.1	69.7	46.0	41.1	71.9	36.1	66.4	55.2	52.1	41.7	147.6	
RetinaNet [42]	80.4	88.0	44.1	78.9	78.7	45.7	85.1	81.3	67.8	56.0	153.5	
Cascade R-CNN [44]	74.5	87.6	57.5	81.2	72.3	60.8	84.3	80.5	69.9	43.6	129.6	
RT-DETR [23]	77.6	89.9	58.5	90.0	59.3	72.4	87.2	80.1	73.2	42.0	136.5	
ViTDet [45]	64.4	85.6	33.7	48.9	81.2	70.1	72.6	79.8	62.2	63.2	178.3	
YOLOv5 [46]	66.4	90.9	53.5	90.3	76.6	80.1	82.0	78.9	73.8	21.1	76.6	
YOLOv7 [33]	74.6	90.1	57.8	81.4	74.6	80.7	83.3	80.2	74.9	24.9	104.7	
YOLOv8 [47]	79.7	91.5	62.1	88.1	76.6	77.0	84.9	81.6	75.6	26.3	77.6	
YOLOX [48]	78.4	91.8	63.1	90.5	70.9	81.9	86.5	82.3	76.7	19.0	76.0	
Super-YOLO [50]	65.4	90.0	59.2	85.0	74.4	70.1	74.1	77.7	70.1	17.7	78.0	
Gold-YOLO [34]	81.7	88.6	64.6	85.0	72.4	78.9	86.0	80.2	74.8	21.5	46.0	
YOLOv11 [49]	77.1	90.5	64.4	91.3	77.1	83.4	86.7	81.9	77.4	23.0	76.9	
MCFM [51]	83.4	90.8	65.1	91.4	78.5	84.6	86.4	80.0	77.6	-	-	
ours	82.4	92.0	65.3	91.6	79.3	86.2	86.3	82.5	78.1	19.5	75.2	

**Table 3:** Quantitative comparison of our algorithm with other models on the HRRSD dataset. (The best results are highlighted in red, the second-best results are highlighted in blue, and the third-best results are highlighted in purple).

Model	AL	B	S	V	BC	BD	CR	GTF	HB	PL	ST	TC	TJ	mAP	Para (M)	FLOPs(G)
Faster R-CNN [43]	86.1	81.4	81.2	83.4	62.7	82.4	89.2	95.6	88.3	45.8	82.9	83.3	70.8	79.5	41.7	147.6
RetinaNet [42]	93.5	87.2	89.8	93.4	67.2	86.1	94.3	98.6	91.6	55.0	94.2	90.7	83.1	86.5	56.0	153.5
CornerNet [52]	91.6	88.1	89.4	89.3	68.9	83.1	90.1	94.6	90.8	60.9	89.3	87.1	78.6	84.8	32.7	86.2
MGCN [53]	93.3	89.8	89.0	89.9	55.8	81.7	92.9	93.5	93.0	64.7	92.1	84.0	79.9	84.6	-	-
ViTDet [45]	87.1	74.4	71.5	80.1	48.3	77.7	88.3	81.3	76.5	49.5	83.8	77.3	69.4	74.2	63.2	178.3
YOLOv5 [46]	98.1	92.8	92.2	95.2	76.4	83.9	91.7	97.4	94.6	65.2	96.2	93.4	80.8	89.1	21.1	76.6
YOLOv7 [33]	98.2	90.7	92	94.9	76.8	82.7	91.7	97.7	94.5	65.8	96.4	93.8	80.3	88.9	24.9	104.7
Super-YOLO [50]	98.4	89.6	88.8	89.3	69.8	83.7	89	95.4	92.4	56.0	94.9	92.2	76.7	85.9	17.7	78.0
Gold-YOLO [34]	98.9	93.3	92.7	94.7	70.5	91.1	94.5	98.1	95.8	67.4	96.3	92.2	84.5	90.0	21.5	46.0
YOLOv11 [49]	98.4	93.0	92.0	88.5	76.7	84.7	89.8	98.9	95.3	67.6	98.0	93.7	83.5	89.2	23.0	76.9
MCFM [51]	98.9	93.5	92.8	95.0	76.9	85.3	93.5	98.7	94.2	66.4	96.9	93.7	84.9	90.1	-	-
ours	99.1	93.7	92.2	94.8	77.1	87.7	95.1	98.9	95.9	67.7	97.2	92.8	85.2	90.6	19.5	75.2

**Table 4:** Quantitative comparison of our algorithm with other models on the RSOD dataset. (The best results are highlighted in red, the second-best results are highlighted in blue, and the third-best results are highlighted in purple).

Model	Playground	Oiltank	Overpass	Aircraft	mAP	Para (M)	FLOPs(G)
Faster R-CNN [43]	77.4	81.0	46.7	73.1	69.6	41.7	147.6
Retina-Net [42]	86.2	83.3	63.7	88.4	80.4	56.0	153.5
Cascade R-CNN [56]	99.0	96.1	83.2	94.2	91.3	43.6	129.6
YOLOv3 [28]	95.1	91.4	77.7	90.6	88.7	28.7	84.1
YOLOv5 [46]	96.7	97.7	84.1	95.6	93.5	21.1	76.6
YOLOv7 [33]	95.9	96.8	84.5	94.0	92.8	24.9	104.7
YOLOv8 [47]	97.0	97.4	88.5	94.8	94.4	26.3	77.6
YOLOv10 [54]	97.6	96.3	69.9	94.9	89.7	15.4	59.1
GAM [55]	98.4	97.0	90.8	97.5	95.9	-	-
ours	97.2	97.5	93.3	98.1	96.5	19.5	75.2

**Table 5:** Ablation Study of the CC-MSFE on the DIOR, HRRSD, and RSOD datasets.

Dataset	Item	mAP50	mAP50:95	APs	APm	APl	Para (M)	FLOPs (G)
DIOR	Without DWConv and Cross-Channel	80.3	63.4	70.5	75.4	85.7	19.0	74.5
	DWConv Without Cross-Channel	82.5	63.1	71.2	75.3	85.9	19.2	74.6
	Both DWConv and Cross-Channel	86.3	67.0	81.4	85.4	86.1	19.5	75.2
HRRSD	Without DWConv and Cross-Channel	84.3	62.8	63.8	68.2	75.2	19.0	74.5
	DWConv Without Cross-Channel	85.8	64.3	66.1	70.4	75.8	19.2	74.6
	Both DWConv and Cross-Channel	92.2	68.5	69.3	73.2	78.3	19.5	75.2
RSOD	Without DWConv and Cross-Channel	91.1	63.2	25.3	73.2	70.3	19.0	74.5
	DWConv Without Cross-Channel	93.3	65.3	26.7	74.4	71.0	19.2	74.6
	Both DWConv and Cross-Channel	96.8	69.7	29.5	77.5	73.5	19.5	75.2

**Table 6:** Ablation study of the CSCANet on the DIOR, HRRSD, and RSOD datasets.

Dataset	CSCANet			mAP50	mAP50:95	APs	APm	APl	Para (M)	FLOPs (G)
	CA	SA	CAFM							
DIOR		√	√	83.4	65.3	78.5	79.1	85.5	19.0	75.1
	√		√	84.2	65.7	77.4	80.2	85.3	18.8	74.9
	√	√		82.5	62.5	75.8	76.4	84.2	20.5	75.9
	√	√	√	86.3	67.0	81.4	85.4	86.1	19.5	75.2
HRRSD		√	√	88.9	65.6	67.3	70.3	77.1	19.0	75.1
	√		√	88.3	66.2	67.5	71.0	77.8	18.8	74.9
	√	√		87.1	63.5	65.1	68.4	76.1	20.5	75.9
	√	√	√	92.2	68.5	69.3	73.2	78.3	19.5	75.2
RSOD		√	√	93.1	66.1	28.1	75.2	73.0	19.0	75.1
	√		√	93.6	66.4	27.9	75.0	73.1	18.8	74.9
	√	√		91.9	65.7	25.0	72.8	72.6	20.5	75.9
	√	√	√	96.8	69.7	29.5	77.5	73.5	19.5	75.2

**Table 7:** Performance comparison of CSCA with various attention mechanisms on the DIOR, HRRSD, and RSOD datasets.

Dataset	Attention	mAP50	mAP50:95	APs	APm	APl	Para(M)	FLOPs(G)
DIOR	SK	83.8	65.3	75.1	80.3	78.9	28.2	85.3
	CA	85.6	65.9	78.2	84.0	84.2	18.8	68.4
	Wavelet Attention	85.9	66.5	76.3	84.2	85.2	20.3	76.1
	CSCA(ours)	86.3	67.0	81.4	85.4	86.1	19.5	75.2
HRRSD	SK	90.2	67.8	67.3	70.3	75.3	28.2	85.3
	CA	91.8	68.1	69.1	71.6	77.9	18.8	68.4
	Wavelet Attention	92.0	68.3	68.7	72.1	78.0	20.3	76.1
	CSCA(ours)	92.2	68.5	69.3	73.2	78.3	19.5	75.2
RSOD	SK	95.2	68.7	27.4	75.2	70.9	28.2	85.3
	CA	95.9	69.3	28.7	77.0	72.1	18.8	68.4
	Wavelet Attention	96.5	69.6	28.1	76.7	72.4	20.3	76.1
	CSCA(ours)	96.8	69.7	29.5	77.5	73.5	19.5	75.2

## Data Availability

The data associated with this research are available online. The DIOR dataset is available for download at https://opendatalab.org.cn/OpenDataLab/DIOR (accessed on 2 December 2022). The RSOD dataset is available for download at https://github.com/CrazyStoneonRoad/TGRS-HRRSD-Dataset?tab=readme-ov-file (accessed on 15 January 2023) The RSOD dataset is available for download at https://github.com/RSIA-LIESMARS-WHU/RSOD-Dataset- (accessed on 12 December 2023).

## Abbreviations

The following abbreviations are used in this manuscript:

RSOD	Object Detection in Remote Sensing Images
CC-MSFE	Cross-Channel Multi-Scale Feature Extraction
CSCA	Channel-Spatial Cross-Attention Mechanism
CA	Channel Attention
SA	Spatial Attention
CAFM	Cross-Attention Fusion Module
BN	Batch Normalization
ConvFFN	Convolutional Feed-Forward Network
MLP	Multilayer Perceptron
